# Endoscopic Ultrasound Fine-Needle Biopsy versus Fine-Needle Aspiration for Tissue Sampling of Abdominal Lymph Nodes: A Propensity Score Matched Multicenter Comparative Study

**DOI:** 10.3390/cancers13174298

**Published:** 2021-08-26

**Authors:** Antonio Facciorusso, Stefano Francesco Crinò, Nicola Muscatiello, Paraskevas Gkolfakis, Jayanta Samanta, Juliana Londoño Castillo, Christian Cotsoglou, Daryl Ramai

**Affiliations:** 1 Gastroenterology Unit, Department of Surgical and Medical Sciences, University of Foggia, 71122 Foggia, Italy; antonio.facciorusso@virgilio.it (A.F.); nmuscatiello@ospedaliriunitifoggia.it (N.M.); 2 Gastroenterology and Digestive Endoscopy Unit, Department of Medicine, The Pancreas Institute, University Hospital of Verona, 37134 Verona, Italy; stefanofrancesco.crino@aovr.veneto.it; 3 Hepatopancreatology and Digestive Oncology Unit, Department of Gastroenterology, CUB Erasme Hospital, Université Libre de Bruxelles (ULB), 1070 Brussels, Belgium; paraskevas.gkolfakis@bordet.be; 4 Department of Gastroenterology, Post Graduate Institute of Medical Education and Research, Chandigarh 160012, India; dj_samanta@yahoo.co.in; 5 Gastroenterology Unit, University Ces, Calle 56, Medellin 050021, Colombia; clondono.juliana@uces.edu.co; 6 General Surgery Department, ASST-Vimercate, 20871 Vimercate, Italy; christian.cotsoglou@asst-brianza.it; 7 Division of Gastroenterology and Hepatology, University of Utah, Salt Lake City, UT 84132, USA

**Keywords:** endoscopic ultrasound, fine-needle aspiration, fine-needle biopsy, colorectal cancer, metastases, pancreatic cancer

## Abstract

**Simple Summary:**

Lymph node (LN) sampling or staging is crucial to the management of malignancies. The use of endoscopic ultrasound for lymph node sampling can be performed with EUS fine-needle aspiration (FNA) or EUS fine-needle biopsy (FNB). However, it remains unclear whether EUS-FNA or EUS-FNB is superior for sampling of abdominal lymph nodes. In this study, we retrospectively compared a large volume of patients who underwent lymph node sampling using EUS-FNA or EUS-FNB. Most patients were diagnosed with metastatic colorectal disease. We found that EUS-FNB had a higher diagnostic accuracy and sensitivity compared to EUS-FNA. Both modalities had no adverse events. Thus, the results support the use of EUS-FNB for abdominal lymph node sampling.

**Abstract:**

There is a paucity of evidence on the comparison between endoscopic ultrasound (EUS) fine-needle biopsy (FNB) and fine-needle aspiration (FNA) for lymph node (LNs) sampling. The aim of this study was to compare these two approaches in a multicenter series of patients with abdominal tumors. Out of 502 patients undergoing EUS sampling, two groups following propensity score matching were compared: 105 undergoing EUS-FNB and 105 undergoing EUS-FNA. The primary outcome was diagnostic accuracy. Secondary outcomes were diagnostic sensitivity, specificity, sample adequacy, optimal histological core procurement, number of passes, and adverse events. Median age was 64.6 years, and most patients were male in both groups. Final diagnosis was LN metastasis (mainly from colorectal cancer) in 70.4% of patients in the EUS-FNB group and 66.6% in the EUS-FNA group (*p* = 0.22). Diagnostic accuracy was significantly higher in the EUS-FNB group as compared to the EUS-FNA group (87.62% versus 75.24%, *p* = 0.02). EUS-FNB outperformed EUS-FNA also in terms of diagnostic sensitivity (84.71% vs. 70.11%; *p* = 0.01), whereas specificity was 100% in both groups (*p* = 0.6). Sample adequacy analysis showed a non-significant trend in favor of EUS-FNB (96.1% versus 89.5%, *p* = 0.06) whereas the histological core procurement rate was significantly higher with EUS-FNB (94.2% versus 51.4%; *p* < 0.001). No procedure-related adverse events were observed. These findings show that EUS-FNB is superior to EUS-FNA in tissue sampling of abdominal LNs.

## 1. Introduction

Assessing lymph nodes (LNs) involvement from a neoplastic disease has a fundamental impact on tumor staging and treatment. However, lymphadenopathy commonly represents a diagnostic challenge for the clinician. Other disease states can present with lymphadenopathy, including benign conditions, such as infections and inflammatory disease [1]. Accurate imaging-guided LN sampling is therefore important to ascertain the underlying diagnosis and to enable adequate clinical management. Among these techniques, endoscopic ultrasound (EUS)-guided sampling plays a pivotal role in the diagnostic management of thoracic and particularly of abdominal LNs and has currently superseded more invasive methods, such as mediastinoscopy or laparotomy [2].

Endosonographic characteristics of malignant lymph nodes include a large size, hypoechogenicity, distinct border, round shape, and high tissue stiffness on elastography [3,4]. Unfortunately, simple LN morphology assessed through EUS is not sufficient to definitively distinguish benign nodes from malignant ones, thus an appropriate tissue sampling-aimed technique with concomitant pathological confirmation is often employed [5,6].

The diagnostic accuracy of EUS-guided fine-needle aspiration (EUS-FNA) of LNs is not very high, especially if compared to other solid masses, such as pancreatic tumors or solid liver lesions, and particularly poor in the case of lymphomas [7,8,9]. In fact, a recent meta-analysis showed a pooled sensitivity with EUS-FNA of 87%, increased to 91% in the presence of rapid on-site cytologic evaluation (ROSE) [10].

In the last years, a paradigm shift towards increased use of fine-needle biopsy (FNB) was observed in clinical practice [11]. The promising results observed with newer FNB needles, such as the Franseen needle (Acquire^®^ [Boston Scientific, Marlborough, MA, USA]) and the Fork-tip needle (SharkCore^®^ [Medtronic, Dublin, Ireland]), in tissue acquisition of pancreatic masses and of subepithelial lesions require confirmation also in the setting of LN biopsy [12,13,14,15,16,17,18].

Only a few studies enrolling both patients with mediastinal and intrabdominal lymphoadenopathy compared these two sampling strategies. However, these studies were limited by a small sample size and single-center design or poor statistical methodology [19,20]. Therefore, the aim of this study was to compare, through a robust propensity-matched design, the diagnostic performance of EUS-FNB in comparison to EUS-FNA in the workup of intraabdominal lymphadenopathies in a multicenter cohort of patients.

## 2. Results

### 2.1. Patients

The baseline characteristics of the whole study population of 352 patients (105 undergoing EUS-FNB and 247 EUS-FNA) initially enrolled in the study are reported in Table 1. The number of cases enrolled in the two study arms in each center are reported in Table S1. Mean age was 64.4 ± 7 in the EUS-FNB group and 66.3 ± 5 in the EUS-FNA group (*p* = 0.6). Male patients represented the majority of subjects in both groups (*p* = 0.26). Peri-hepatic and peri-pancreatic were the most common locations of target LNs, with 48 (45.7%) and 27 (25.7%) patients in the EUS-FNB group and 93 (37.6%) and 82 (33.1%) patients in the EUS-FNA group, respectively (*p* = 0.36).

Mean lesion size was significantly larger in the EUS-FNA group (32.4 ± 0.8 mm versus 21.4 ± 2.1 mm in the EUS-FNB group; *p* = 0.04). Most of the punctures were performed through the trans-duodenal route in both groups (*p* = 0.30), mainly with a 22 G needle (71.4% and 62.7% in the two groups, respectively; *p* = 0.11). A similar number of patients were on antithrombotic therapy, with no difference between the two groups (32.3% in the EUS-FNB group and 38% in the EUS-FNA group, *p* = 0.31). The median follow-up in nonresected patients was 14.6 ± 3 months.

Study outcomes observed before propensity score matching are reported in Table S2. Diagnostic sensitivity (*p* = 0.03), accuracy (*p* = 0.04), and histological core procurement rate (*p* < 0.001) were significantly superior in patients undergoing EUS-FNB as compared to EUS-FNA.

**Figure 1:**
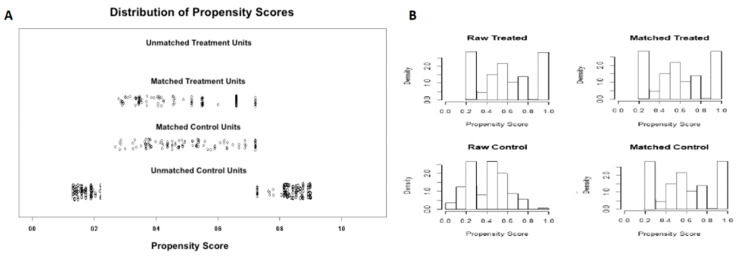
Results of propensity score matching. (**A**) Propensity score matching jitter plot. (**B**) Propensity score matching histogram.

After 1-to-1 propensity score matching, 210 patients were selected for comparison: 105 EUS-FNB and 105 EUS-FNA patients. Details of the propensity score matching are shown in Figure 1A,B.

The characteristics of the 210 propensity score-matched patients are reported in Table 2. Median age was 64.4 ± 7 and 64.6 ± 5 years in the two groups (*p* = 0.9), with 67 (63.8%) and 68 (64.7%) male patients in the EUS-FNB and EUS-FNA group, respectively (*p* = 0.86).

Again, peri-hepatic and peri-pancreatic were the most frequent locations of sampled LNs (45.7% and 46.6% peri-hepatic, and 25.7% and 24.8% peri-pancreatic in the two groups, respectively; *p* = 0.94) and no difference in terms of mean lesion size was observed (21.4 ± 2.1 mm in the EUS-FNB group and 22.4 ± 1.8 mm in the EUS-FNA group, *p* = 0.64). The trans-duodenal approach was preferred in 59 (56.1%) patients in each group whereas the trans-gastric route was used in 31 patients (29.5%) in both groups (*p* = 1.0). Additionally, an equal number of patients were sampled with 22 G in the two groups (*p* = 1.0). Thirty-four patients in the EUS-FNB group (32.3%) and 37 in the EUS-FNA group (35.3%) were on antithrombotic treatment at the time of the procedure. EUS-FNA patients underwent a significantly higher number of passes as compared to the EUS-FNB group (3.2 ± 0.9 versus 2.4 ± 0.9, *p* = 0.03).

In the EUS-FNB group, ProCore^®^ (Cook Medical Inc., Bloomington, IN, USA) was used in 10 patients, SharkCore^®^ in 56 patients, and Acquire^®^ in 49 subjects.

### 2.2. Outcomes

A detailed list of study outcomes is reported in Table 3. The diagnostic accuracy rate was significantly higher in the EUS-FNB group as compared to the EUS-FNA group (87.62% versus 75.24%, *p* = 0.02). Likewise, EUS-FNB outperformed EUS-FNA also in terms of diagnostic sensitivity (84.71% vs. 70.11%; *p* = 0.01), whereas specificity was 100% in both groups (*p* = 0.6).

Sample adequacy analysis showed a non-significant trend in favor of EUS-FNB (96.1% versus 89.5%, *p* = 0.06). On the other hand, the histological core procurement rate was significantly higher with EUS-FNB (94.2% versus 51.4%; *p* < 0.001). Of note, a high false negative rate was observed in the EUS-FNA group, thus explaining the difference between the sample adequacy and accuracy rates registered in these patients.

Final diagnosis was metastasis in 74 patients (70.4%) in the EUS-FNB group and 70 patients in the EUS-FNA group (66.6%), while lymphoma was detected in 20 (19%) and 15 (14.2%) patients, respectively (*p* = 0.22). In particular, metastases from colorectal cancer were diagnosed in 55 patients (52.3%) in the EUS-FNB group and 52 (49.5%) patients in the EUS-FNA group, from pancreatic cancer in 18 patients (17.1%) in the EUS-FNB group and 15 (14.2%) in the EUS-FNA group, and from gastric cancer in 1 patient (0.9%) in the EUS-FNB group and 3 patients (2.8%) in the EUS-FNA group. Sarcoidosis was diagnosed in seven patients (6.6%) in the EUS-FNB group and nine (8.5%) in the EUS-FNA group. No procedure-related adverse events were registered in any of the study groups.

### 2.3. Subgroup Analysis

As described in Table 4, the results of the main analysis were confirmed in the subgroup analysis performed according to LN location (peri-hepatic versus peri-gastric versus peri-rectal). In fact, while sample adequacy was not significantly different (*p* = 0.19, 0.6 and 0.07 in the 3 groups, respectively), diagnostic sensitivity was constantly superior in the EUS-FNB group regardless of the LN location (85.49%, 90%, and 92.3% versus 68.42%, 75%, and 71.4% with EUS-FNA in the 3 different locations, respectively). Similarly, diagnostic accuracy with EUS-FNB ranged from 89.58% in peri-gastric to 93.33% in peri-rectal LNs and it was significantly superior to EUS-FNA (*p* = 0.01 in all of the three subgroups).

The superior performance of EUS-FNB was confirmed also in the subgroup analysis performed according to the needle size (22 G versus 25 G). Of note, sample adequacy was confirmed to be superior in EUS-FNB patients only in the 22 G subgroup (94.6% versus 84%, *p* = 0.03), whereas no significant difference was observed when 25 G needles were used (93.3% versus 80%, *p* = 0.12), although this finding should be interpreted with caution due to the limited sample size in this specific setting.

The results of the main analysis were confirmed in patients with LN metastases, with diagnostic accuracy and sensitivity of 90% and 88.7% in the EUS-FNB group and 75.7% and 74.6% in the EUS-FNA group (*p* = 0.02 and 0.05, respectively).

In patients with lymphoma, the diagnostic performance of EUS-FNA was particularly poor, with accuracy and sensitivity of 60% and 53.8%, respectively. As a consequence, even in this setting, EUS-FNB clearly outperformed EUS-FNA. In patients with benign disease, diagnostic accuracy was 91% (69.3–97.8%) with EUS-FNB and 73% (52.3–86.6%) with EUS-FNA (*p* = 0.03) whereas sensitivity was 89.1% (67.5–98.1%) and 73.8% (65.1–82.5%) with the two techniques, respectively (*p* = 0.04).

## 3. Discussion

While EUS-guided tissue acquisition represents a well-defined and widely recognized diagnostic tool for mediastinal lymphoadenopathy, its role in abdominal LNs sampling is less certain. Recent studies showed that this technique is safe and effective while a prospective series enrolling 142 patients reported rates for sample adequacy up to 91% [21].

However, the reported diagnostic performance with EUS-FNA in this setting was suboptimal, in particular in patients with lymphoma, where for a complete diagnosis including subclassification, a relatively large amount of material may be required. Therefore, the need of morphologic, immunophenotypic, genotypic, and molecular analysis has traditionally pushed hematologists/oncologists to prefer surgical excision, although a large retrospective study showed relatively high sensitivity for diagnosing lymphoma with 19 G FNA needle and subclassification was possible for 91% of the patients [22,23].

However, evidence on EUS-guided tissue acquisition in patients with abdominal lymphoadenopathy remains scarce, particularly the comparison of EUS-FNB versus standard FNA. A recent retrospective multicenter study showed similar sensitivity and accuracy between FNA and FNB (67.21% vs. 75.00%, *p* = 0.216 and 78.80% vs. 83.17%, *p* = 0.423, respectively), although with better specificity with FNB (100.00% vs. 93.62%, *p* = 0.01) [19]. Of note, the diagnostic performance of EUS-FNB was significantly better than FNA in the subgroups of abdominal and peri-hepatic LN locations and ROSE was used in a consistent part of patients undergoing EUS-FNA, thus at least partially explaining the lack of statistical difference observed in the main analysis [19].

A small prospective study enrolling 48 patients also did not find a significant difference between the two sampling strategies, although the diagnostic sensitivity for lymphoma was borderline superior in favor of EUS-FNB performed using the ProCore^®^ needle (*p* = 0.06) [20].

Comparative results based on studies using newer FNB needles are still scarce. In fact, the Franseen and Fork-tip needles, characterized by a surface with multiple cutting points designed to provide improved control at the puncture site and stability at the tip allowing for enhanced penetration, showed very promising results in other abdominal masses [11,12,13,14,15,16,17,18]. However, definitive assumptions of their comparative performance in the case of LN sampling cannot be drawn. In fact, scarce and discording results were published on the performance of these newer FNB needles in this setting. While a Japanese study failed to find a significant difference between the Acquire^®^ needle and FNA, a large single-center American series showed superior results with newer FNB needles even in patients with lymphoadenopathy [24,25].

Therefore, given the scarce and conflicting evidence on this topic, we decided to perform a multicenter retrospective analysis of our series of patients who underwent EUS-FNB compared to EUS-FNA for LN sampling.

To the best of our knowledge, the current manuscript represents the first comparative series specifically conducted in patients with abdominal lymphoadenopathy. In order to overcome the potential biases related to the retrospective nature of the study and to properly take into account all confounding variables, we performed a propensity score matching analysis on the basis of several demographic and lesion-related covariates, thus two perfectly balanced treatment groups were obtained.

The diagnostic accuracy rate was significantly higher in the EUS-FNB group as compared to the EUS-FNA group (87.62% versus 75.24%, *p* = 0.02) and EUS-FNB outperformed EUS-FNA also in terms of diagnostic sensitivity (84.71% vs. 70.11%; *p* = 0.01), whereas, as expected, specificity was 100% in both groups (*p* = 0.6).

The results of our EUS-FNA series are in line with the current literature based on studies not using ROSE [10]. On the other hand, EUS-FNB in our study showed results slightly superior to previous studies, probably due to the prevalent use of newer FNB needles in our series as compared to the large proportion of patients sampled with the reverse bevel needle in previous reports [19,20,23].

Sample adequacy analysis showed a non-significant trend in favor of EUS-FNB (96.1% versus 89.5%, *p* = 0.06), a result consistent with the aforementioned studies [19,20]. As expected, the histological core procurement rate was significantly higher with EUS-FNB (94.2% versus 51.4%; *p* < 0.001) and this might be of particular relevance in patients with suspected lymphoma, where adequate histological samples are needed for molecular diagnosis and subclassification, as shown in the subgroup of lymphoma patients reported in Table 4.

The results of the main analysis were confirmed in the subgroup analysis performed according to LN location, again in line with previous series [19]. Concerning the needle size, most of the patients were sampled with a 22 G needle in both groups, showing better adequacy with the 22 G FNB needle as compared to FNA (*p* = 0.03). On the other hand, no significant difference was observed when 25 G needles were used (93.3% versus 80%, *p* = 0.12), although this finding should be interpreted with caution due to the limited sample size in this specific setting. A larger sample size would have likely shown similar results also with the 25 G needle, as reported in the case of other solid lesions where the diagnostic performance of these newer devices was similar regardless of the needle size [13]. The number of needle passes was considerably lower in the case of EUS-FNB, again in line with the results observed in other abdominal solid lesions [25,26].

Finally, no procedure-related adverse events were reported, thus confirming the excellent safety profile of these procedures even in patients with lymphoadenopathy.

This study has a number of strengths: firstly, it is the first published series directly comparing the two sampling strategies specifically in patients with abdominal lymphoadenopahy. Second, the accurate statistical design and the completeness of the collected data strengthen the results of our analysis. Third, the multicenter nature of the current study represents a guarantee of the reproducibility of our results.

Nevertheless, the paper has some weaknesses. Its main limitation is the retrospective nature of the study, which could have led to selection biases. However, a propensity score matching analysis based on the baseline covariates known to influence diagnostic outcomes was performed in order to obviate to the aforementioned bias. Thus, the study groups were perfectly balanced without statistically different baseline parameters. Another limitation is the fact that cost considerations were beyond the scope of the present study and could not be addressed. Finally, in non-resected patients, the final diagnosis was established based on the clinical and radiological evolution of the disease. Therefore, despite the follow-up time being longer than 12 months in these groups of patients, false-negative biopsy results could be misdiagnosed as true negative, especially in those cases who underwent chemotherapy.

## 4. Materials and Methods

### 4.1. Patients

From a multicenter prospectively collected database of consecutive patients undergoing EUS-guided sampling of abdominal LNs in 6 high-volume centers between 2012 and 2021, we retrospectively reviewed data from 502 patients. Institutional Review Board (IRB) approval for this study was obtained.

The following exclusion criteria were used: (1) age < 18 years; (2) clear indication to surgical treatment; and (3) coagulopathy (international normalized ratio > 1.5, platelets < 50,000). All endoscopic procedures were performed by board-certified 10-year-experienced gastroenterologists. Antibiotic prophylaxis was not used routinely before the procedure and patients in antithrombotic treatment suspended the anticoagulant/antiaggregant agent and underwent bridging therapy with enoxaparin according to current guidelines [27].

After excluding patients who did not meet inclusion criteria, we analyzed two groups of patients: 247 patients who underwent EUS-FNA before 2016 and 105 treated with EUS-FNB from 2016 onward.

### 4.2. Procedures

A linear array echoendoscope (Pentax FG-36UA or Pentax EG3870-UTK, Pentax Europe, Ltd., Hamburg, Germany) was used for all EUS-guided tissue sampling procedures under deep sedation with propofol (Diprivan^®^, AstraZeneca, London, UK) administered by an anesthesiologist [28].

EUS-FNA was conducted with 22 G or 25 G (EchoTip Ultra, Cook Medical Inc., Bloomington, IN, USA) whereas EUS-FNB was performed using 22 G or 25 G Acquire^®^, SharkCore^®^, or ProCore^®^ needles (Cook Medical Inc., Bloomington, IN, USA). ROSE was not available in any of the centers involved in the study. No predefined protocol was used in the study and the type of suction or use of stylet was left to the choice of the single operator.

**Figure 2:**
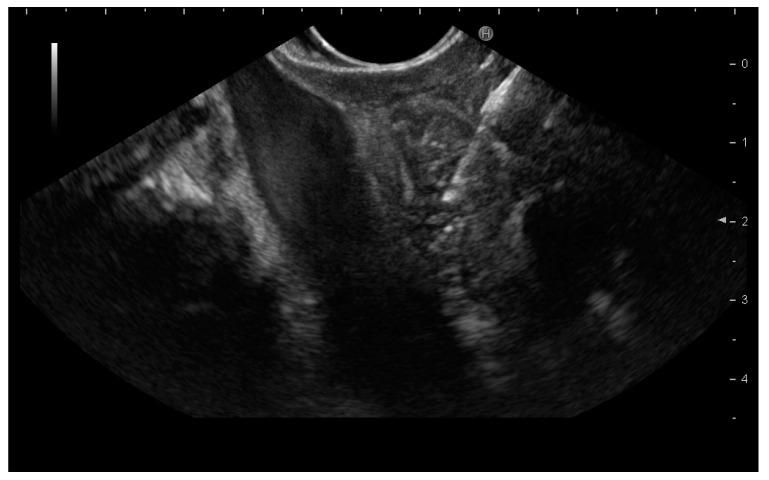
Endoscopic ultrasound-guided lymph node sampling.

In general, after lesions were identified and punctured under EUS guidance, a fanning technique was performed. More than 10 to and fro movements were made within the lesion and at least 2 passes were performed (Figure 2). After being grossly checked for adequacy, samples were prepared for cytological examination or histological assessment. Eventual additional passes were performed when macroscopic assessment raised concerns on the adequacy of the sample. The pathologists evaluating the specimen were blinded to the method adopted for EUS-tissue acquisition.

A board-certified anesthesiologist continuously monitored patients during the procedure with an automated noninvasive blood pressure device, electrocardiogram tracing, and pulse oximetry. Depending on the complexity of the procedure in addition to patient comorbidities, patients were hospitalized and observed for 24 h or discharged the same day of the procedure. In both cases, the monitoring protocol was the same.

### 4.3. Outcomes

The primary outcome was diagnostic accuracy, defined as the summary of true positives (TPs) + true negatives (TNs) on the total number of patients. The gold standard for diagnosis was considered surgery or the evolution of the disease assessed for at least 6 months by a combination of clinical course and/or imaging studies [29].

Additional outcomes were diagnostic sensitivity (proportion of positives correctly identified with the test (TPs) on the prevalence of disease in the study cohort), diagnostic specificity (proportion of negatives correctly identified as such (TNs) among the patients who were not affected by the disease in the study cohort), and sample adequacy (proportion of samples considered sufficient for diagnosis) [29]. Additional outcomes were optimal histologic core procurement, which was defined as the proportion of patients with samples adequate for histological diagnosis, number of needle passes needed to obtain adequate samples, and adverse event rate. Atypical cells were considered as negative, whereas cases “suspicious of malignancy” were considered as positive for malignancy. A severe adverse event was defined as one that required hospitalization, was life-threatening, or resulted in death or disability.

### 4.4. Statistical Analysis

Categorical variables were reported as the number of cases and percentage, and differences between groups were compared using Chi-square and McNemar analyses before and after matching, respectively.

Continuous variables were expressed as the mean and standard deviation and differences between groups were explored by the Mann–Whitney and Wilkoxon-rank test before and after matching, respectively. All analyses were 2-tailed, and the threshold of significance was assessed at ≤0.05.

To overcome biases owing to the different distribution of covariates among patients who were submitted to EUS-guided tissue acquisition with FNB or FNA, a 1-to-1 match was created using propensity score analysis.

The propensity score represents the probability of each individual patient being assigned to a particular condition in a study given a set of known covariates [30].

A multivariate logistic regression was built to predict the probability of each individual patient being submitted to the two groups on the basis of several demographic and lesion-related covariates, namely age, gender, LN location, lesion size, diagnostic sample approach, needle size, and antithrombotic therapy.

The predictive values were then used to obtain a 1-to-1 match by using the nearest neighbor matching within a specified caliper distance. Nearest neighbor matching within a specified caliper distance selects for matching an untreated subject whose propensity score is closest to that of the treated subject (“nearest neighbor matching” approach) with the further restriction that the absolute difference in the propensity scores of matched subjects must be below some pre-specified threshold (the caliper distance) [31,32]. Thus, patients for whom the propensity score could not be matched because of a greater caliper distance were excluded from further analysis. As suggested by Austin, a caliper of width equal to 0.2 of the standard deviation of the logit of the propensity score was used, as this value has been found to minimize the mean squared error of the estimated treatment effect [31].

Subgroup analysis according to LN location (peri-hepatic versus peri-pancreatic versus peri-rectal), needle used (22 G versus 25 G), and final diagnosis (metastasis versus lymphoma) was also performed. The statistical analysis was run using the MatchIt package in R Statistical Software 3.0.2 (Foundation for Statistical Computing, Vienna, Austria).

## 5. Conclusions

Our analysis provides robust evidence on the comparison between EUS-FNB and EUS-FNA in patients with abdominal lymphoadenopathy. Based on our findings, EUS-FNB should be preferred to standard EUS-FNA for sampling intraabdominal LNs in patients with suspected malignancy.

## Figures and Tables

**Table 1:** Baseline patients’ characteristics before propensity score matching.

Variable	EUS-FNB (*n* = 105)	EUS-FNA (*n* = 247)	*p* Value
Age (years)	64.4 ± 7	66.3 ± 5	0.6
Gender			
M	67 (63.8%)	142 (57.4%)	0.26
F	38 (36.2%)	105 (42.6%)	-
Lymph node location			
Peri-gastroduodenal	12 (11.4%)	24 (9.7%)	-
Peri-hepatic	48 (45.7%)	93 (37.6%)	-
Peri-pancreatic	27 (25.7%)	82 (33.1%)	0.36
Celiac	3 (2.8%)	15 (6%)	-
Peri-rectal	15 (14.4%)	33 (13.6%)	-
Lesion size (mm)	21.4 ± 2.1	32.4 ± 0.8	**0.04**
Diagnostic sample approach			
Trans-gastric	31 (29.5%)	94 (38%)	-
Trans-duodenal	59 (56.1%)	120 (48.4%)	0.30
Trans-rectal	15 (14.4%)	33 (13.6%)	-
Needle size			
22 G	75 (71.4%)	155 (62.7%)	0.11
25 G	30 (28.6%)	92 (37.3%)	-
Antithrombotic therapy	34 (32.3%)	94 (38%)	0.31

Continuous variables are reported as mean values and standard deviations. Comparisons were performed with the Mann–Whitney U test for continuous variables and Fisher exact test for categorical ones. The following demographic and lesion-related variables were selected for propensity score calculation: age, gender, lymph node location, lesion size, diagnostic sample approach, needle size, antithrombotic therapy. Abbreviations: FNA, fine-needle aspiration; FNB, fine-needle biopsy. Significances are reported in bold.

**Table 2:** Baseline characteristics after propensity score matching.

Variable	EUS-FNB (*n* = 105)	EUS-FNA (*n* = 105)	*p* Value
Age (years)	64.4 ± 7	64.6 ± 5	0.9
Gender			
M	67 (63.8%)	68 (64.7%)	0.86
F	38 (36.2%)	37 (35.3%)	-
Lymph node location			
Peri-gastroduodenal	12 (11.4%)	12 (11.4%)	-
Peri-hepatic	48 (45.7%)	49 (46.6%)	-
Peri-pancreatic	27 (25.7%)	26 (24.8%)	0.94
Celiac	3 (2.8%)	3 (2.8%)	-
Peri-rectal	15 (14.4%)	15 (14.4%)	-
Lesion size (mm)	21.4 ± 2.1	22.4 ± 1.8	0.64
Diagnostic sample approach			
Trans-gastric	31 (29.5%)	31 (29.5%)	-
Trans-duodenal	59 (56.1%)	59 (56.1%)	1.0
Trans-rectal	15 (14.4%)	15 (14.4%)	-
Needle size			
22 G	75 (71.4%)	75 (71.4%)	1.0
25 G	30 (28.6%)	30 (28.6%)	-
Number of passes	2.4 ± 0.9	3.2 ± 0.9	**0.03**
Antithrombotic therapy	34 (32.3%)	37 (35.3%)	0.21

Continuous variables are reported as mean values and standard deviations. Comparisons were performed with the Mann–Whitney U test for continuous variables and Fisher exact test for categorical ones. The following demographic and lesion-related variables were selected for propensity score calculation: age, gender, lymph node location, lesion size, diagnostic sample approach, antithrombotic therapy. Abbreviations: FNA, fine-needle aspiration; FNB, fine-needle biopsy. Significances are reported in bold.

**Table 3:** Study outcomes comparing endoscopic ultrasound fine-needle aspiration and fine-needle biopsy.

Outcome	EUS-FNB	EUS-FNA	*p* Value
-	(105 pts)	(105 pts)	**-**
Sensitivity	84.71% (75.2–91.6%)	70.11% (59.3–79.4%)	**0.01**
Specificity	100% (83.16–100%)	100% (81.4–100%)	0.6
Diagnostic adequacy	101 (96.1%)	94 (89.5%)	0.06
Diagnostic accuracy	92	79	-
-	87.62% (79.7–93.2%)	75.24% (65.8–83.1%)	0.02
Final diagnosis			
Metastasis	74 (70.4%)	70 (66.6%)	
Lymphoma	20 (19%)	15 (14.2%)	0.22
Benign	7 (6.6%)	9 (8.5%)	
Inconclusive	4 (4%)	11 (10.7%)	
Histological core procurement	99 (94.2%)	54 (54.4%)	**<0.001**
Procedure-related adverse events	0 (0%)	0 (0%)	1.0

Values are expressed as number (percentage) and 95% confidence intervals. Abbreviations: FNA, fine-needle aspiration; FNB, fine-needle biopsy. Significances are reported in bold.

**Table 4:** Subgroup analysis according to lymph node location, needle used, and final diagnosis.

Outcome	EUS-FNB	EUS-FNA	*p* Value
**Peri-Hepatic Location**			
	**EUS-FNB (48 patients)**	**EUS-FNA (49 patients)**	
Sensitivity	86.49% (71.2–95.4%)	68.42% (51.3–82.5%)	**0.01**
Specificity	100% (71.5–100%)	100% (71.5–100%)	0.7
Diagnostic adequacy	45 (93.7%)	42 (85.7%)	0.19
Diagnostic accuracy	89.58% (77.3–96.5%)	75.51% (61.1–86.6%)	**0.01**
Histological core procurement	44 (91.6%)	24 (48.9%)	**<0.001**
**Peri-pancreatic location**			
	**EUS-FNB (27 patients)**	**EUS-FNA (26 patients)**	
Sensitivity	90% (68.3–98.7%)	75% (50.9–91.3%)	**0.02**
Specificity	100% (59–100%)	100% (54–100%)	0.5
Diagnostic adequacy	25 (92.59%)	23 (88.4%)	0.6
Diagnostic accuracy	92.59% (75.7–99%)	80.77% (60.6–93.4%)	**0.01**
Histological core procurement	23 (85.1%)	15 (57.7%)	**<0.001**
**Peri-rectal location**			
	**EUS-FNB (15 patients)**	**EUS-FNA (15 patients)**	
Sensitivity	92.3% (63.9–99.8%)	71.4% (41.9–91.6%)	**0.03**
Specificity	100% (15.8–100%)	100% (2.5–100%)	0.56
Diagnostic adequacy	15 (100%)	13 (86.6%)	0.07
Diagnostic accuracy	93.33% (68–99.8%)	73.33% (44.9–92.2%)	**0.01**
Histological core procurement	13 (86.6%)	6 (40%)	**<0.001**
**22 G needle**			
	**EUS-FNB (75 patients)**	**EUS-FNA (75 patients)**	
Sensitivity	91.6% (81.6–97.2%)	74.6% (62.5–84.4%)	**0.01**
Specificity	100% (78.2–100%)	100% (63–100%)	0.6
Diagnostic adequacy	71 (94.6%)	63 (84%)	**0.03**
Diagnostic accuracy	93.33% (85.1–97.8%)	77.33% (66.2–86.2%)	**0.02**
Histological core procurement	67 (89.3%)	31 (41.3%)	**<0.001**
**25 G needle**			
	**EUS-FNB (30 patients)**	**EUS-FNA (30 patients)**	
Sensitivity	88% (68.7–97.4%)	75% (55.1–89.3%)	**0.04**
Specificity	100% (47.8–100%)	100% (15.8–100%)	0.8
Diagnostic adequacy	28 (93.3%)	24 (80%)	0.12
Diagnostic accuracy	90% (73.4–97.9%)	76.67% (57.7–90%)	**0.03**
Histological core procurement	25 (83.3%)	12 (40%)	**<0.001**
**Metastases**			
	**EUS-FNB (74 patients)**	**EUS-FNA (70 patients)**	
Sensitivity	88.7% (78.1–95.3%)	74.6% (62.5–84.4%)	**0.05**
Specificity	100% (63–100%)	100% (29.2–100%)	0.6
Diagnostic adequacy	69 (93.2%)	57 (81.4%)	0.66
Diagnostic accuracy	90% (80.4–95.8%)	75.7% (64–85.7%)	**0.02**
Histological core procurement	63 (85.1%)	30 (42.8%)	**<0.001**
**Lymphoma**			
	**EUS-FNB (20 patients)**	**EUS-FNA (15 patients)**	
Sensitivity	88.2% (63.5–98.5%)	53.8% (25.1–80.8%)	**0.008**
Specificity	100% (29.2–100%)	100% (15.8–100%)	0.3
Diagnostic adequacy	18 (90%)	12 (80%)	0.4
Diagnostic accuracy	90% (68.3–98.7%)	60% (32.3–83.6%)	**0.006**
Histological core procurement	17 (85%)	4 (26.6%)	**<0.001**
**Benign disease**			
	**EUS-FNB (7 patients)**	**EUS-FNA (9 patients)**	
Sensitivity	89.1% (67.5–98.1%)	73.8% (65.1–82.5%)	**0.04**
Specificity	100% (29.2–100%)	100% (15.8–100%)	0.3
Diagnostic adequacy	7 (100%)	9 (100%)	1.0
Diagnostic accuracy	91% (69.3–97.8%)	73% (52.3–86.6%)	**0.03**
Histological core procurement	7 (100%)	2 (22.2%)	**<0.001**

Values are expressed as number (percentage) and 95% confidence intervals. Abbreviations: FNA, fine-needle aspiration; FNB, fine-needle biopsy. Significances are reported in bold.

## Data Availability

The data presented in this study are available on request from the corresponding author. The data are not publicly available due to confidentiality and ethical reasons.

## Supplementary Materials

The following are available online at https://www.mdpi.com/article/10.3390/cancers13174298/s1, Table S1: Number of cases enrolled in each center, Table S2: Study outcomes comparing endoscopic ultrasound fine needle aspiration and fine needle biopsy before propensity score matching.
