# Supplementary material for: Endoscopic Ultrasound Fine-Needle Biopsy versus Fine-Needle Aspiration for Tissue Sampling of Abdominal Lymph Nodes: A Propensity Score Matched Multicenter Comparative Study
Source: Cancers (Basel). 2021 Aug 26;13(17):4298. doi: 10.3390/cancers13174298 (PMC8428361; doi:10.3390/cancers13174298)
Supplement: Supplementary file 1 [file cancers-13-04298-s001.zip › cancers-1320789-supplementary.pdf]

cancers

MDPI

Supplementary Material: Endoscopic Ultrasound Fine-Needle Biopsy versus Fine-Needle Aspiration for Tissue Sampling of Abdominal Lymph Nodes: A Propensity Score Matched Multicenter Comparative Study

Antonio Facciorusso, Stefano Francesco Crinò, Nicola Muscatiello, Paraskevas Gkolfakis, Jayanta Samanta, Juliana Londoño Castillo, Christian Cotsoglou and Daryl Ramai

Table S1. Number of cases enrolled in each center.

Center	EUS-FNB (*n* = 105)	EUS-FNA (*n* = 247)
1	20	45
2	15	40
3	14	38
4	17	36
5	16	48
6	23	40

Table S2. Study outcomes comparing endoscopic ultrasound fine needle aspiration and fine needle biopsy before propensity score matching.

Variable	EUS-FNB (105 pts)	EUS-FNA (247 pts)	*P* Value
Sensitivity	84.71% (75.2%-91.6%)	73.18% (61.5%-80.4%)	**0.03**
Specificity	100% (83.16%-100%)	100% (81.4%-100%)	0.6
Diagnostic adequacy	101 (96.1%)	222 (89.8%)	0.08
Diagnostic accuracy	87.62% (79.7%-93.2%)	77.21% (68.8%-84.5%)	**0.04**
Histological core procurement	99 (94.2%)	121 (48.9%)	**<0.001**
Procedure-related adverse events	0 (0%)	0 (0%)	1.0

Values are expressed as number (percentage) and 95% confidence intervals. Abbreviations: FNA, fine-needle aspiration; FNB, fine-needle biopsy.
